# *Helicobacter pylori* and Atrial Fibrillation: Insights into Their Inter-Relationship

**DOI:** 10.31083/RCM26911

**Published:** 2025-04-18

**Authors:** Weiting Feng, Qiming Liu, Shenghua Zhou, Mingxian Chen, Yichao Xiao

**Affiliations:** ^1^Department of Cardiovascular Medicine, Second Xiangya Hospital, Central South University, 410011 Changsha, Hunan, China; ^2^Department of Cardiovascular Medicine, Sun Yat-Sen Memorial Hospital, Sun Yat-Sen University, 510120 Guangzhou, Guangdong, China

**Keywords:** *Helicobacter pylori*, atrial fibrillation, inflammation, gut microbiota

## Abstract

*Helicobacter pylori* (*H. pylori*) infection and atrial fibrillation (AF) are prevalent global health concerns that significantly impact societal and economic well-being. This study explored the potential associations between *H. pylori* infection and the incidence and progression of AF. Emerging research suggests that *H. pylori* may influence AF through various pathways, including systemic inflammation, metabolic disturbances, immune responses, and changes in the gut microbiota. These pathways provide a novel perspective on the etiology of AF, suggesting that chronic *H. pylori* infection could exacerbate or even initiate the arrhythmic events typical of AF. Current evidence, while preliminary, points to significant correlations, particularly through changes in markers such as C-reactive protein (CRP) and lipid metabolism, which are heightened in individuals with active *H. pylori* infection. However, the exact mechanisms and causal nature of this relationship remain elusive, with studies showing conflicting results. This inconsistency underscores the need for more comprehensive and rigorously designed clinical and experimental research to elucidate fully the interactions between *H. pylori* infection and AF. Understanding these connections is crucial for developing innovative treatments and management strategies targeting microbial influences in AF patients. Future research should focus on defining the role of *H. pylori* eradication in the clinical management of AF assessing its impact on disease progression and patient outcomes.

## 1. Introduction

*Helicobacter pylori* (*H. pylori*), a flagellated, spiral-shaped, Gram-negative, microaerophilic bacterium, represents the most prevalent chronic bacterial infection globally [1]. The unique morphology and physiological capabilities of this bacterium allow it to penetrate the gastric mucosa and colonize the interstitial space between the mucinous sodium carbonate barrier, thereby allowing it to survive the direct effects of gastric acid. By producing urease to break down urea, *H. pylori* generates CO_2_ and ammonia to neutralize stomach acid and create a slightly alkaline environment suitable for survival [1, 2]. Statistically, approximately 43.1% of the global population is infected with *H. pylori*, with prevalence rates varying significantly across different regions—reaching as high as 56.1% in the Eastern Mediterranean and 53.3% in Africa [3, 4]. In addition, some studies have reported that the infection rate of *H. pylori* is approximately 30% in developed countries and up to 80% in some developing countries. The disease incidence in adults is significantly greater than in children [5]. *H. pylori* infection often presents asymptomatically but can lead to upper gastrointestinal diseases, such as gastritis and gastric ulcers. Furthermore, chronic *H. pylori* infection can result in serious complications, including gastric cancer and mucosa-associated lymphoid tissue (MALT) lymphoma, if not properly treated [6, 7, 8]. The high infection rate and pathogenicity have placed a great burden on society.

Atrial fibrillation (AF), the most common persistent cardiac arrhythmia encountered clinically, is predominantly diagnosed via electrocardiogram and is characterized by rapid and irregular atrial rhythms [9]. According to a study on the 2019 Global Burden of Disease Database, the total number of global patients with atrial fibrillation/atrial flutter (AF/AFL) reached 59.7 million in 2019, with 315,000 deaths due to AF and 8.39 million disability-adjusted life years (DALYs) lost, highlighting the significant harm that AF promotes as a global public health problem [10]. The incidence of AF varies across age groups; for example, it affects up to 9% of the population over 65 years of age and nearly one in five individuals over 85 [11, 12]. Major risk factors for AF include age, hypertension, diabetes, and obesity, all of which are closely linked with severe complications such as stroke, heart failure, and premature death [13].

In recent years, an increasing body of research has explored the potential link between *H. pylori* infection and AF. Given that both *H. pylori* and AF are significant global public health concerns, understanding their interplay is crucial for revealing the mechanisms of disease onset and developing novel preventive and therapeutic strategies [1, 9, 12]. Although primarily affecting gastric health, *H. pylori* and cardiac-affecting AF may seem unrelated, recent studies suggest a possible connection. However, the exact mechanisms of this potential link are not fully understood [14, 15, 16, 17]. The preliminary findings indicate common pathways involving inflammation, metabolic disorders, immune responses, and changes in the gut microbiota [17, 18]. This review aims to systematically evaluate the relationship between *H. pylori* and AF, explore the potential pathogenic mechanisms, assess current diagnostic and therapeutic approaches, and discuss the implications of this relationship for patient prognosis. We hope to pave the way for future research through an in-depth analysis, particularly in developing targeted treatment and prevention strategies. Thus, this study elaborates on the relationship between *H. pylori* and AF, aiming to provide deeper insights and guidance for clinical practice.

## 2. *Helicobacter pylori* and Atrial Fibrillation: Exploring Connections

*H. pylori *is commonly believed to be closely associated with stomach-related diseases. Meanwhile, further investigations into *H. pylori* have revealed associations with additional gastric disorders, including neurological, hematological, respiratory, and cardiovascular diseases [19, 20, 21, 22, 23, 24]. The cardiovascular diseases related to *H. pylori* include AF, stroke, coronary heart disease, hypertension, and atherosclerosis [25]. The relationship between *H. pylori* and AF has always been a research focus, as shown in Table 1 (Ref. [14, 16, 17, 18, 26, 27, 28, 29, 30, 31, 32, 33, 34, 35, 36]).

**Table 1.** **Correlation studies between *H. pylori* and atrial fibrillation**.

Author (year)	Brief description of study	Conclusions and results
Montenero *et al*. [17] (2005)	The study recruited 30 patients with paroxysmal AF and 29 patients with persistent AF to explore the relationship between *H. pylori* and AF.	A highly significant correlation was found between AF and *H. pylori*, which was especially strong in patients with persistent AF.
Badran and Mahfouz [30] (2007)	A study recruited 185 patients with coronary heart disease to investigate the relationship between *H. pylori* and AF among these patients.	A significant link was found between AF and *H. pylori* Cag A-positive strains in patients with coronary heart disease.
Bunch *et al*. [29] (2008)	The study involved 943 patients with AF.	An association between *H. pylori* and AF was observed, but this association weakened after adjusting for other factors, especially age.
Platonov *et al*. [31] (2008)	The study recruited 72 permanent AF patients and 72 healthy individuals from the same region to explore the effects of *Chlamydia pneumoniae* and *H. pylori* on idiopathic permanent AF.	Permanent AF was associated with elevated CRP levels, but this elevation was unrelated to early infection with *Chlamydia pneumoniae* and *H. pylori*.
Lunetta *et al*. [35] (2009)	A 7-year prospective study included 120 *H. pylori*-positive patients and 60 *H. pylori* serum-negative patients.	No significant difference was found in the incidence of AF between the two groups, and inflammation caused by *H. pylori* was ruled out as a cause of AF.
Ki *et al*. [32] (2010)	The study recruited 60 patients with AF (including 27 patients with paroxysmal AF and 33 with persistent AF) while using 36 patients with other rapid arrhythmias as controls.	*H. pylori* infection was associated with a decrease in serum TGF-β1 levels, which may lead to the recurrence of AF.
Schimke *et al*. [36] (2010)	The study involved 1179 patients with type 2 diabetes to explore whether the Cag A of *H. pylori* is related to the pathological changes in major blood vessels.	The *Cag A* gene in *H. pylori* was not associated with coronary heart disease, peripheral vascular disease, cerebrovascular disease, and AF.
Xie *et al*. [33] (2014)	In total, 600 patients were recruited to study the risk factors and corresponding intervention measures for *H. pylori* infection in AF patients.	Ages over 65 years and not receiving *H. Pylori* treatment are risk factors for *H. pylori* infection in patients with AF.
Geng *et al*. [26] (2014)	Recruited 45 AF patients and 45 health inspectors to investigate *H. pylori* in AF patients and the correlation between *H. pylori* infection and blood–lipid indicators.	*H. pylori* infection is not associated with AF, but *H. pylori* infection can promote the occurrence and development of AF by affecting lipid levels.
Wang *et al*. [16] (2015)	In total, 585 patients with AF were recruited to explore the relationship between *H. pylori* infection and different types of AF.	The values of *H. pylori* in patients with long-standing AF were significantly higher than those in short-standing AF and control groups. *H. pylori* δ value ≥4% remained an independent predictor for long-standing AF.
Yan *et al*. [27] (2016)	A meta-analysis was conducted by searching PubMed, Embase, Web of Science, and the Cochrane Library databases for research on the relationship between *H. pylori* and arrhythmia.	*H. pylori* infection is one of the risk factors for AF in Asia and Africa.
Zhang *et al*. [18] (2018)	In total, 86 AF patients and 65 healthy examinees were selected to study *H. pylori*. The correlation between *H. pylori* infection and AF, CRP, and lipid metabolism.	*H. pylori* infection may affect the occurrence and development of AF through lipid metabolism and inflammatory response pathways.
Tetta *et al*. [34] (2019)	The review included six retrospective studies exploring the correlation between AF and *H. pylori*, including 335 *H. pylori*-positive AF patients, 621 *H. pylori*-negative AF patients, 643 *H. pylori*-positive normal individuals, and 1322 *H. pylori* negative normal individuals.	According to data analysis, no significant correlation was found between *H. pylori* infection and AF.
Rivington and Twohig [28] (2020)	A retrospective study based on IBM Explories (1999–2019) quantified the risk of AF-related diseases.	The likelihood of developing AF after infection with *H. pylori* increases, but the magnitude of the increase is minimal.
Farah *et al*. [14] (2024)	In total, 180 patients with *H. pylori* were recruited for the study.	AF is associated with *H. pylori* and is more significantly associated with age and elevated CRP levels in *H. pylori* patients.

AF, atrial fibrillation; *H. pylori*, *Helicobacter pylori*; CRP, C-reactive protein; Cag A, cytotoxin-associated gene A; TGF-β1, transforming growth factor-beta1.

The association between *H. pylori* and AF was first proposed by Montenero *et al*. in 2005 [17], who suggested that inflammation mediated by *H. pylori* might play a role. Further studies have highlighted a stronger correlation between AF and *H. pylori*, especially in individuals harboring cytotoxin-associated gene A (Cag A)-positive strains of the bacterium. This relationship suggests a potential underlying mechanism that may involve the generation of autoantibodies [17]. Additionally, *H. pylori* can influence the progression of AF by affecting lipid metabolism pathways [26]. Indeed, *H. pylori* has been identified as an independent contributor in chronic and enduring cases of AF and is acknowledged as a significant risk factor for AF, particularly in regions such as Asia and Africa [27].

However, most studies utilize *H. pylori* IgG (which represents previous *H. pylori* infections). Meanwhile, few studies have investigated the associations between AF and different types of *H. pylori*, which limits the effectiveness of the support for a correlation between the two [17]. A study in China aimed to address these limitations by utilizing Hp values instead of Hp IgG values and investigated the correlation between different types of AF and *H. pylori* [16]. The correlation between different types of AF and *H. pylori* was stronger in long-term persistent AF and permanent AF, which also supports the influence of chronic inflammation caused by *H. pylori* on the occurrence and development of AF [16]. However, these studies were mostly case‒control studies; thus, they lacked more convincing randomized controlled trials and ignored the impact of proton pump inhibitors [37].

Although many studies have confirmed the correlation between *H. pylori* and AF, the validity of this conclusion still needs to be questioned owing to experimental design and regional differences; meanwhile, some studies have also reported a lack of correlation between *H. pylori* and AF. The conclusion that *H. pylori* is an independent factor for AF in Europe and North America is not significant and may be influenced by socioeconomic level [27]. A large retrospective study exploring the risk factors for AF, regarding noninflammatory diseases, hyperthyroidism, chronic obstructive pulmonary disease, alcohol, pulmonary embolism, inflammatory diseases, chronic kidney disease, inflammatory bowel disease, and *H. pylori*, revealed that *H. pylori* had the smallest contribution to AF. However, owing to the retrospective nature of the research data and the inability to obtain individualized data on diagnosis and risk factors, more research is still required to distinguish the correlation between *H. pylori* and AF [28]. Some studies suggest that the relationship becomes less apparent when adjusting for confounders such as age, while others have highlighted that age is a more significant factor in AF [14, 29]. The body of research is still in its exploratory phase, with inconsistent results both domestically and internationally, reflecting the impact of study design, sample selection, and geographical and ethnic differences on research outcomes [16, 18, 28, 29, 30, 31, 32, 33, 35, 36]. Establishing a causal relationship between *H. pylori* and AF, rather than just an association, remains challenging and requires further high-quality clinical research. Thus, conducting multiregional research, expanding sample sizes, conducting randomized controlled trials, and prospective studies in the future can elucidate this relationship and increase the persuasiveness of conclusions.

## 3. Potential Mechanisms of *Helicobacter pylori*-Induced Atrial Fibrillation

The new guidelines divide AF into four stages: Stage 1 refers to a state that poses a risk of causing AF; Stage 2 is the pre-stage of AF, during which patients are prone to AF; Phase 3 includes paroxysmal AF (3A), persistent atrial fibrillation (3B), long-term persistent atrial fibrillation (3C), and successful atrial fibrillation ablation (3D); Phase 4 is permanent AF. The guidelines define AF as a persistent state of the disease [38, 39, 40]. The triggering factors and maintenance mechanisms are the basis for the occurrence and development of different types of AF. Among them, the triggering factors of AF include abnormal electrical activity of pulmonary veins and other cardiac structures, autonomic nervous system disorders, and inflammation. The maintenance mechanism of AF includes multiple episodes, focal triggering theory, inflammation, and atrial remodeling. Early atrial remodeling manifests as electrical remodeling, such as changes in ion channels, whereas late-stage remodeling manifests as structural remodeling, such as tissue fibrosis [41, 42, 43].

**Fig. 1.**
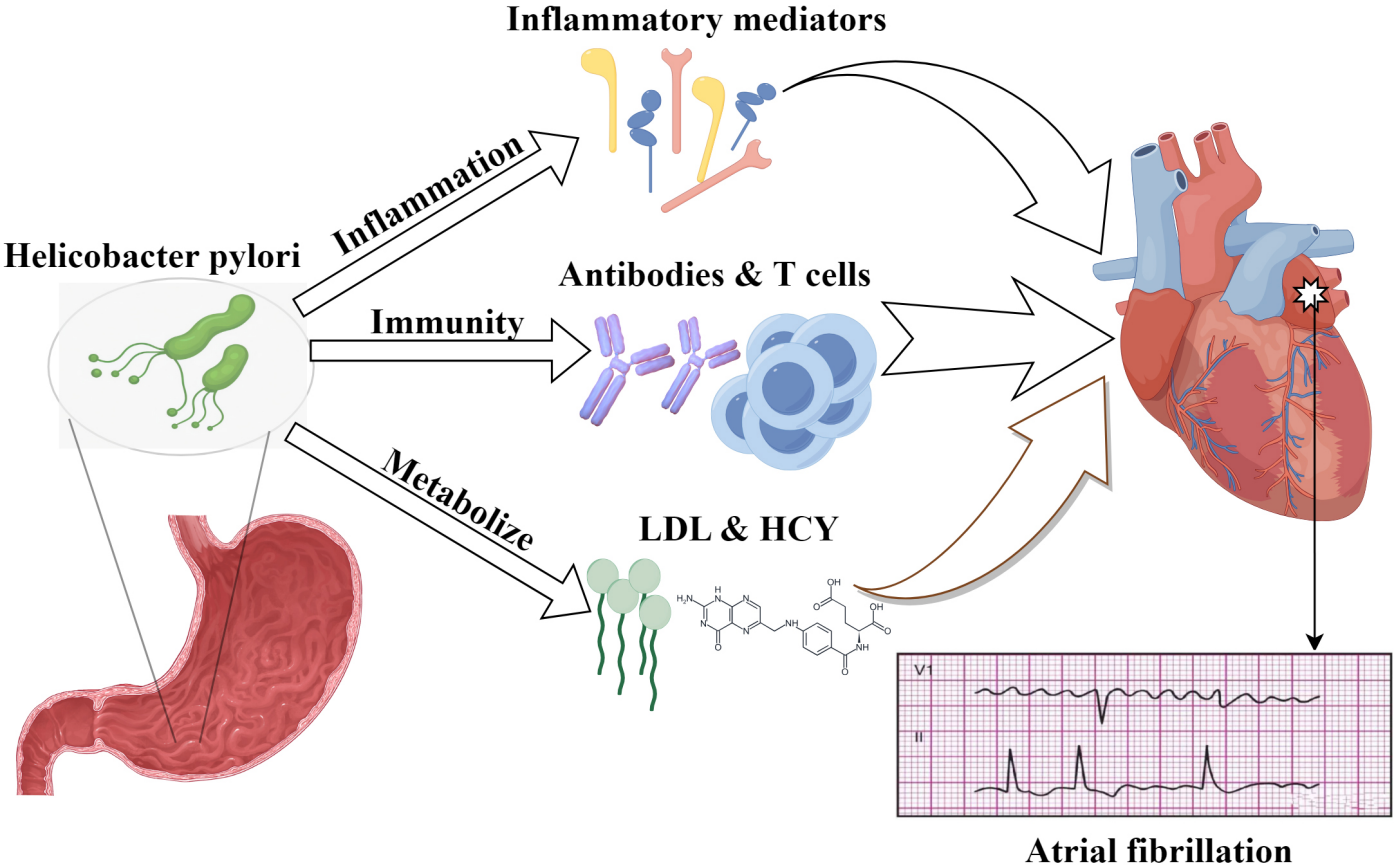
**Possible mechanisms of *H. pylori*-induced AF**. This diagram illustrates the complex interplay between LDL, HCY, and other factors in the context of *H. pylori* infection, which may culminate in AF. LDL, low density lipoprotein; HCY, homocysteine. The figure is created by Figdraw.

Although there is currently no research directly elucidating the specific mechanism through which *H. pylori* affects the occurrence and development of different types of AF, with continued research on the relationship between *H. pylori* and AF, many scholars have proposed hypotheses on how *H. pylori* is involved in the onset and progression of AF. Research has shown that *H. pylori* may directly or indirectly affect AF through inflammation, metabolic disorders, and immune pathways [14, 17, 18, 44], as illustrated in Fig. 1.

### 3.1 Inflammatory Mechanisms

Numerous recent studies have identified a strong association between AF and inflammation, as evidenced by significant correlations between this arrhythmia and various inflammatory biomarkers, including C-reactive protein (CRP), tumor necrosis factor (TNF), and interleukins, interleukin (IL)-1, IL-2, IL-6, and IL-8 [45, 46]. This linkage has led researchers to search for factors that initiate inflammation, with chronic bacterial infection emerging as a likely candidate to trigger and sustain the inflammatory process. Notably, bacterial infections have been increasingly implicated in the pathogenesis of AF, with *H. pylori* garnering particular interest [46].

Studies have shown that *H. pylori* infection can induce gastric and esophageal inflammation and systemic and vascular inflammation, potentially increasing the risk of AF through increased CRP levels [14]. This result is achieved through the activation of the classical complement pathway and the binding of CRP and phosphatidylcholine, the latter of which can cause membrane dysfunction, leading to abnormal sodium and calcium processing. For example, CRP increases the risk of AF by increasing calcium influx through the inward L-type calcium channel in the atrial muscle [47, 48]. Moreover, the Cag A protein produced by *H. pylori* stimulates gastric epithelial cells to secrete the inflammatory mediator IL-8, inducing neutrophil infiltration and consequently causing atrial muscle damage. The extent of the myocardial damage is directly proportional to the duration of infection [16, 28]. Furthermore, research indicates that *H. pylori* immunoglobulin G (representing past infection) is not associated with the duration of AF, suggesting that long-term chronic inflammation caused by *H. pylori* may underlie the development of persistent and permanent AF [16]. Therefore, inflammation may be the basis for triggering and maintaining short-term AF. In contrast, long-term inflammation and the cardiac structural and electrical remodeling caused by inflammation may be among the causes of long-term AF [48].

**Fig. 2.**
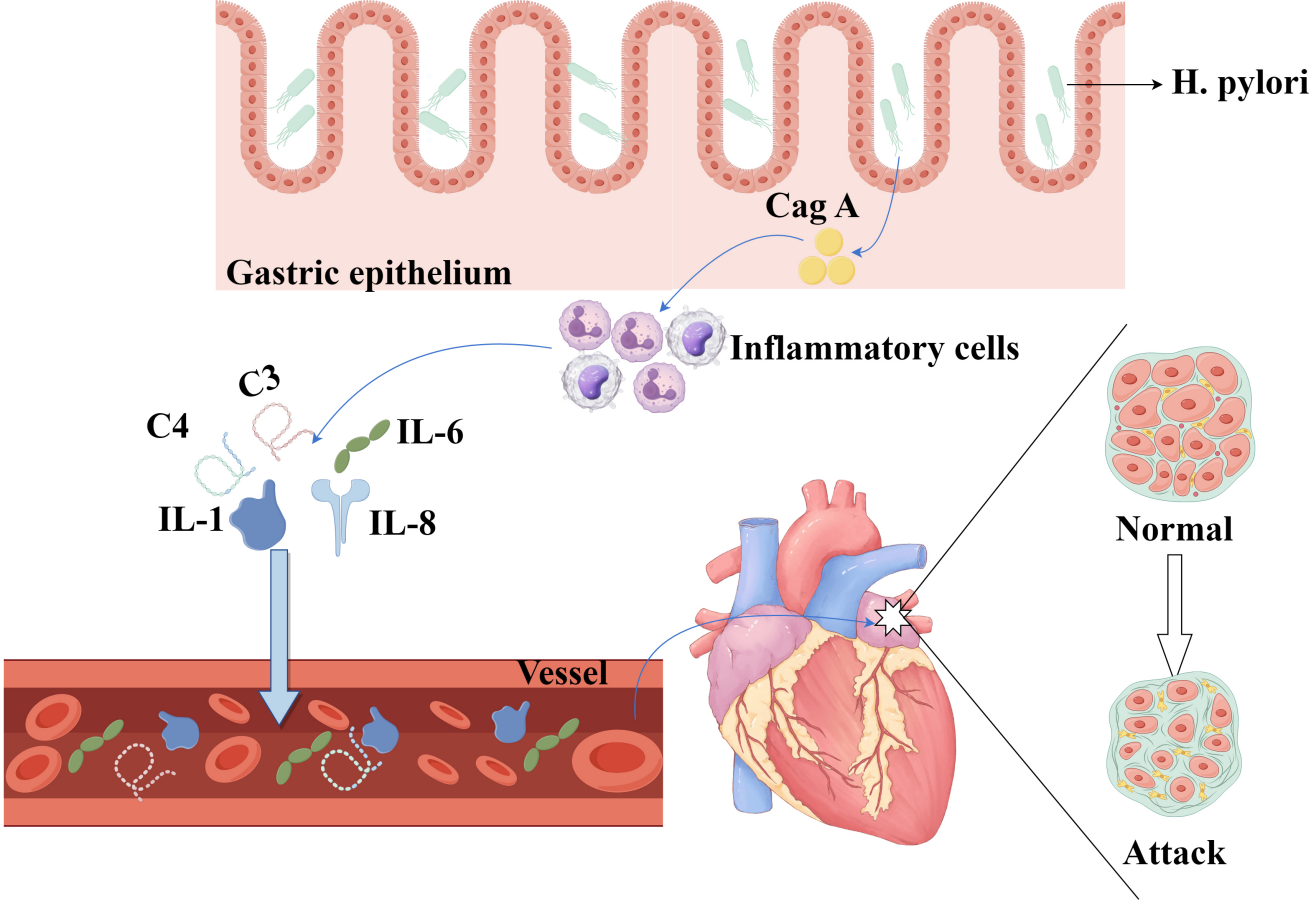
**Inflammatory mechanism of *H. pylori*-induced AF**. The complex inflammatory pathways implicated in *H. pylori*-induced AF highlight the roles of Cag A and inflammatory mediators, such as IL-1, IL-6, IL-8, and complement system components. IL, interleukin. The figure is created by Figdraw.

Additionally, *H. pylori* infection can also cause an increase in other proinflammatory factors, whereby elevated levels of IL-6 and TNF-α due to *H. pylori* infection can promote the progression of AF and even lead to AF-related complications [15, 49]. For example, studies have shown that increases in IL-6 and TNF-α may damage the heart and affect the function of ion channels through two pathways, oxidative stress and calcium mishandling, thereby affecting the occurrence and development of AF [50, 51]. In addition, IL-6 also stimulates the production of effector type 17 helper T cells, leading to IL-17-mediated myocardial fibrosis. The inflammatory mediators involved in *H. pylori*-induced AF also include IL-1β and the complement components C3 and C4 [52], as illustrated in Fig. 2. Homocysteine (HCY), a significant marker of inflammation, contributes to oxidative stress, endothelial damage, and thrombogenesis, potentially initiating and accelerating the process of atherosclerosis, which promotes the onset and progression of AF. Furthermore, HCY can also induce atrial myocyte apoptosis and interstitial fibrosis, leading to atrial remodeling and impacting the development and progression of AF [53, 54].

### 3.2 Metabolic Mechanisms

*H. pylori* infection can disrupt lipid metabolism by generating lipid peroxides and low-density lipoprotein (LDL), which may initiate and accelerate the development of atherosclerosis, thereby contributing to the pathogenesis of AF [18, 55]. Notably, as we will explore further, the gut microbiota modulates these processes and may act as a mediator in establishing the dyslipidemic profile observed in patients with *H. pylori* infection. In support of this hypothesis, antibiotic treatment aimed at eradicating *H. pylori* has been shown to reduce total and LDL cholesterol levels and increase high-density lipoprotein cholesterol (HDL-C) levels [46]. In addition, studies have shown that this process is initiated by chronic inflammation caused by *H. pylori* and is mediated by the inflammatory mediators IL-1, IL-6, and TNF-α [56]. Endothelial cells and macrophages are important for atherosclerosis since endothelial cells can express toll like receptor (TLR)-4, TLR2, and CD14 to recognize *H. pylori* antigens, and macrophages can also express TLR4. The changes in lipid metabolism caused by *H. pylori* and the expression of macrophage-related factors suggest a potential relationship between *H. pylori*, lipids, and atherosclerosis; that is, *H. pylori* may cause disorders of lipid metabolism, leading to the occurrence and development of atherosclerosis. In addition, the increase in lipid peroxides can cause the continuous activation of platelets *in vivo*, which may contribute to thrombosis in atherosclerosis and accelerate the outcome of atherosclerosis [25, 57].

Studies have suggested that *H. pylori* infection may disrupt the absorption of essential nutrients such as vitamin B6, vitamin B12, and folic acid, leading to their deficiency and affecting the normal methylation of homocysteine. This disruption can result in elevated serum homocysteine levels, which may subsequently cause endothelial damage, thrombosis, and atherosclerosis, thus promoting the development and progression of AF [18, 58, 59]. Furthermore, *H. pylori* infection might lead to metabolic syndrome, triggering AF [49, 60, 61], a process that could be associated with the action of galectin-3 [15].

### 3.3 Immune Mechanisms

*H. pylori* acts both as an inflammatory agent and an immune regulator. *H. pylori* infection changes antibody profiles and immune system responses. One notable immune response involves the creation of antibodies against gastric epithelial hydrogen potassium ATPase, which shares structural similarity with the sodium‒potassium ATPase found in cardiac cells, both of which feature a 35 kDa glycoprotein component. Antibodies produced against the hydrogen potassium ATPase during *H. pylori* infection can inadvertently target the cardiac sodium–potassium ATPase, leading to cardiac cell damage and excessive intracellular sodium accumulation [17].

**Fig. 3.**
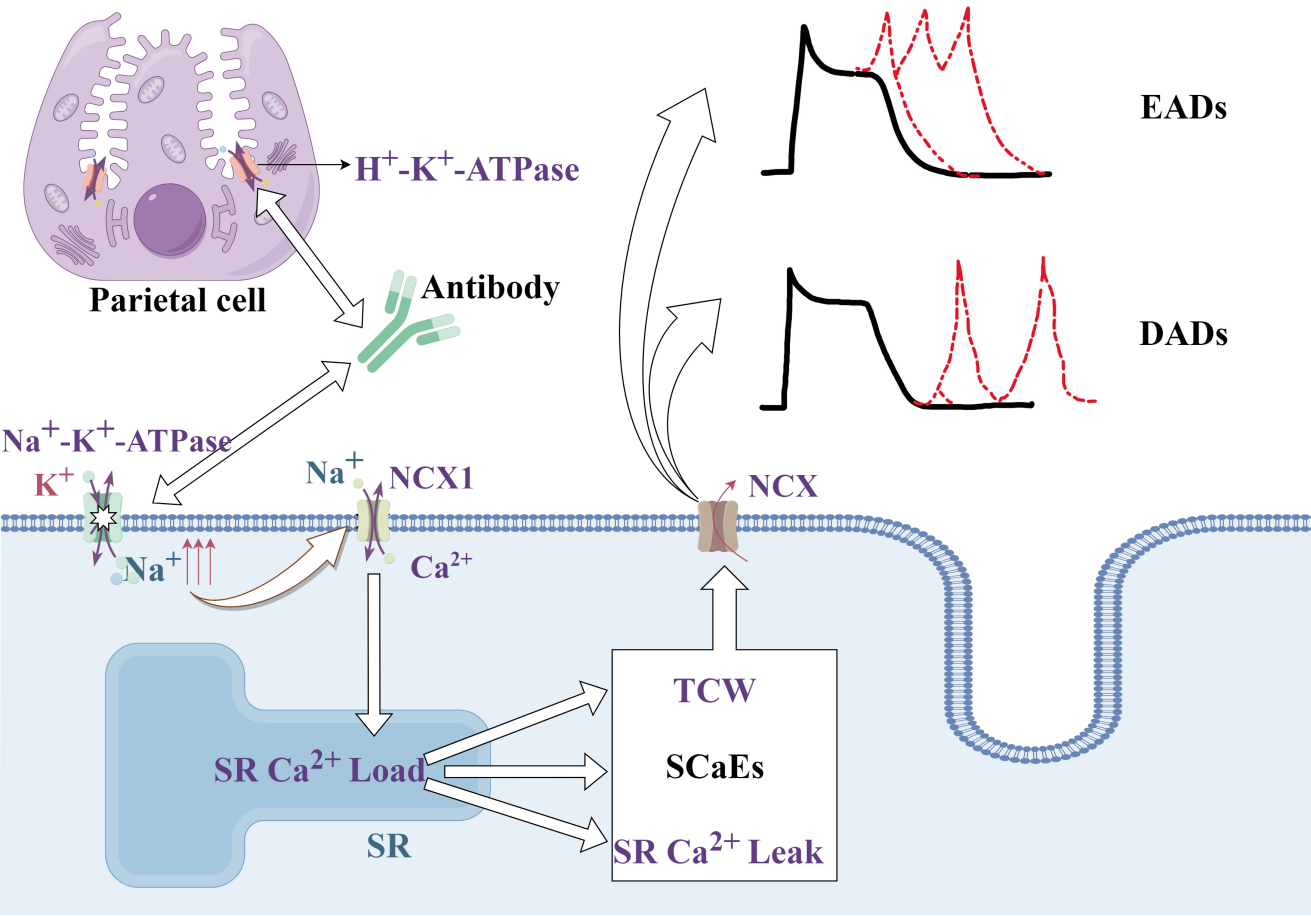
**Mechanism through which antibodies produced by *H. pylori* lead to AF**. The pathway through which antibodies related to *H. pylori* infection can lead to atrial fibrillation, emphasizing the role of altered ion handling and ectopic activity in cardiac cells, which contributes to arrhythmogenesis. EADs, early afterdepolarizations; DADs, delayed after-depolarizations; NCX, Na^+^/Ca^2+^ exchanger; TCW, triggered Ca^2+^ waves; SCaEs, SR Ca^2+^-release events; SR, sarcoplasmic reticulum. The figure is created by Figdraw.

This increase in intracellular sodium can increase the sarcoplasmic reticulum (SR) Ca^2+^ load due to impaired Ca^2+^ extrusion via Na^+^/Ca^2+^ exchanger type-1 (NCX1), which is typically inhibited in conditions such as those induced by cardiac glycosides. An overload of SR Ca^2+^ can also trigger L-type Ca^2+^-current-dependent triggered Ca^2+^ waves (TCW), increased SR Ca^2+^ leakage, and spontaneous SR Ca^2+^-release events (SCaEs). These SCaEs and TCWs activate a transient inward current mediated by the Na^+^/Ca^2+^ exchanger (NCX), resulting in delayed after-depolarizations (DADs) and early after-depolarizations (EADs). Such ectopic activities mediated by DADs and EADs lay an essential groundwork for the occurrence and development of AF [28, 62, 63, 64, 65], as illustrated in Fig. 3.

In the immediate aftermath of cardiac injury, T helper 1 (Th1) cells predominate within the myocardium and are known for their antifibrotic functions by releasing mediators that counteract the profibrotic effects of transforming growth factor-beta (TGF-β). As the injury transitions into a chronic phase, Th2 cells, known for their significant profibrotic activities, become the dominant CD4+ phenotype within the myocardial tissue. Unlike Th1 cells, Th2 cells enhance collagen secretion by directly activating TGF-β or recruiting monocytes to the injury site, thus exacerbating fibrosis [66].

**Fig. 4.**
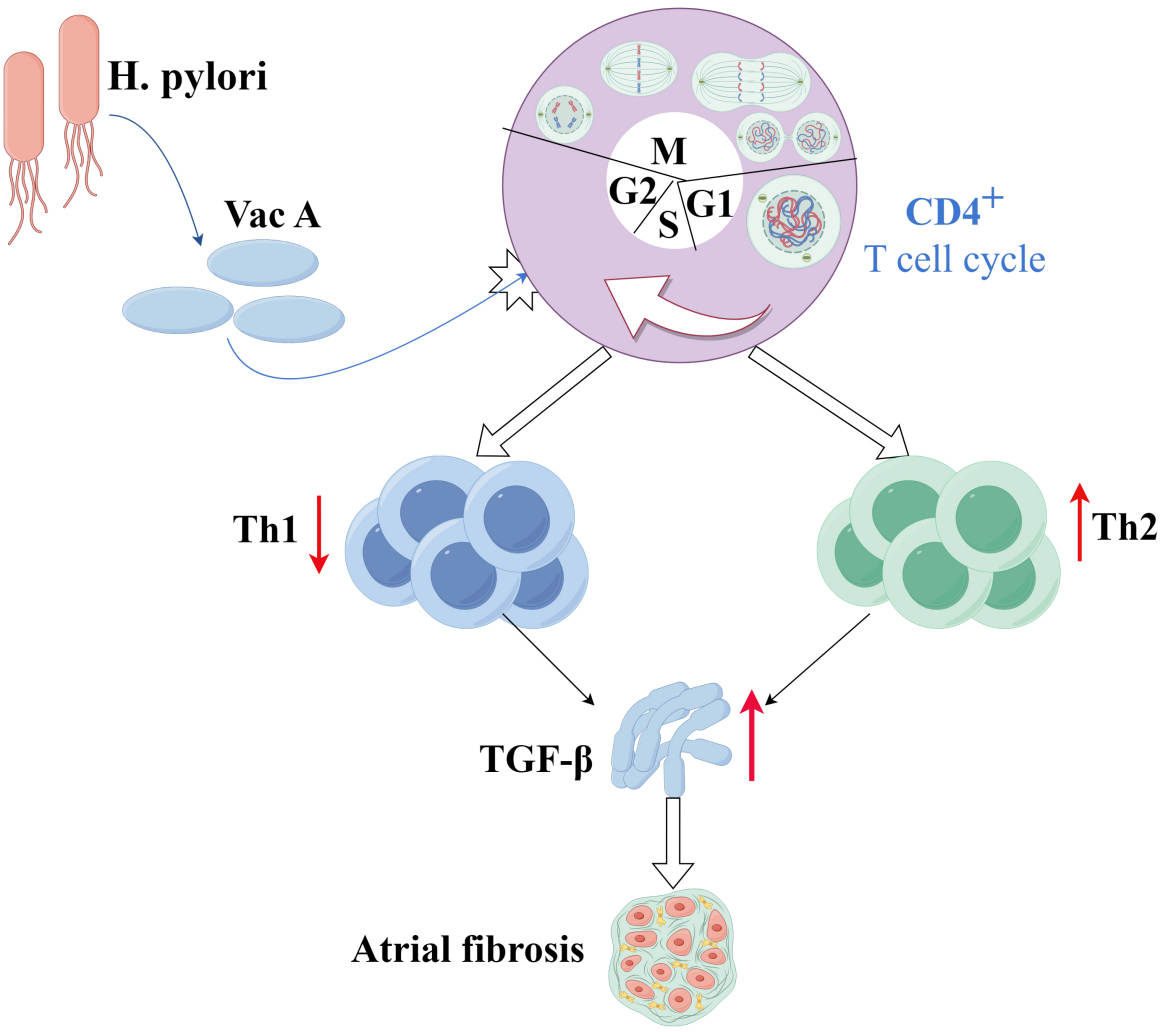
**Mechanism through which *H. pylori* affects the Th1/Th2 balance that leads to AF**. Details of the complex interplay between *H. pylori* infection and immune cell dynamics within the myocardium highlight how changes in the Th1/Th2 balance due to Vac A may lead to increased fibrosis and subsequent atrial fibrillation. Th1, T helper 1 cell; Th2, T helper 2 cell; Vac A, vacuolating cytotoxin A. The figure is created by Figdraw.

By further compounding this dynamic, the virulence factor vacuolating cytotoxin A (Vac A) from *H. pylori* has been shown to block T cell proliferation effectively by inducing G1/S cell cycle arrest. This disruption can shift the Th cell balance from Th1 toward Th2, thereby suppressing Th1 activity and potentially impairing the function of regulatory T cells. Such immunological shifts could increase the risk of AF [27], as illustrated in Fig. 4. Additionally, the increase in TNF-α levels, coupled with the suppression of TGF-β1 mediated by *H. pylori* infection, may further heighten the risk of AF [32]. Moreover, macrophage activation triggered by *H. pylori* can also contribute to the development and progression of AF [49].

### 3.4 Dysregulation of the Gut Microbiota

The gut‒brain axis (GBA) forms a multifaceted network in which the central nervous system (CNS) and the enteric nervous system (ENS) engage in two-way communication via neural, hormonal, metabolic, and immune pathways [67, 68, 69]. The concept of this axis has been expanded to include the microbiota‒gut‒brain axis, recognizing the vital influence of the gut microbiota on functional brain‒gut interactions and their contribution to the onset of diverse diseases [70].

The brain is a pivotal hub in this dynamic network, managing and disseminating information across the enteric, sympathetic, and autonomic nervous systems [22, 68]. *H. pylori *modifies the gut microbial landscape by altering factors such as gastric acidity, immune responses, and the production of antimicrobial peptides and virulence factors. These changes are crucial in shaping gastric and systemic health conditions [71].

*H. pylori* may disrupt the gut microbiota, thereby causing imbalances in the gut‒brain axis, which could lead to dysregulation of the autonomic nervous system. This dysregulation is implicated in the onset and progression of atrial fibrillation, suggesting a novel pathway through which *H. pylori* influences cardiac function indirectly via its effects on the gut microbiota [34, 71, 72, 73, 74].

## 4. Diagnosis of Atrial Fibrillation Associated with *H. pylori*

Electrocardiograms (ECGs) and echocardiograms form the primary diagnostic tools for AF and are essential for visualizing the electrical and structural activity of the heart [65]. Although the specific mechanisms underlying AF are not fully understood, and the pathways through which *H. pylori* might lead to AF are still under investigation, current research suggests potential biomarkers that could assist in diagnosing and predicting AF progression [14].

A study revealed a causal relationship between IL-6 and the occurrence of postoperative AF, which is caused by effects on calcium currents and the stimulation of atrial fibrosis [75, 76, 77]. Another study revealed that IL-17A can represent changes in cardiac fibrosis and left heart function, and it can serve as an independent diagnostic factor to determine the probability of AF recurrence within the first 3 months after catheter ablation [78].

Inflammation plays a central role in the mechanisms through which *H. pylori* is hypothesized to influence AF. These findings suggest the utility of inflammatory markers such as CRP, IL-1, and IL-6 in exploring the etiology and progression of AF and distinguishing between different AF types. However, these markers are not specific to AF and are primarily indicative rather than diagnostic [79].

Additionally, the serum level of homocysteine, an inflammatory marker, may serve as a valuable prognostic indicator for patients with AF [58]. Furthermore, research has indicated that galectin-3, which is related to *H. pylori*, might serve as a potential auxiliary diagnostic marker for AF [15]. Moreover, although highly specific, antibodies such as anti-Cag A and anti-Vac A have an uncertain correlation with AF, limiting their clinical diagnostic value [1, 80]. While these biomarkers offer new perspectives for the diagnosis and management of AF, their application must be considered within the overall clinical context of the patient, and their effectiveness and reliability need further validation in future studies.

## 5. Treatment of Atrial Fibrillation in Relation to *H. pylori*

The fundamental principles of AF treatment aim to eliminate predisposing factors, restore and maintain sinus rhythm, control the ventricular rate, and prevent embolic complications and the recurrence of AF. Treatment modalities are diverse and include pharmacological, electrical, and surgical interventions. Meanwhile, although there is no direct evidence that eradicating *H. pylori* can treat AF, the potential link between the two cannot be overlooked in managing this type of arrhythmia. Furthermore, although no clinical trials have directly studied the benefits and possible risks of eradicating *H. pylori* in the treatment of AF, the potential link between the two cannot be ignored when treating this type of arrhythmia. Theoretically, strategies that suppress inflammation, reduce the production of autoantibodies, adjust metabolic pathways, and restore balance to the gut microbiota might improve the progression of AF [16, 55, 79, 80]. For example, a study suggested that landiolol can reduce the recurrence of AF after esophageal surgery by lowering IL-6 levels [81]. Other studies have shown that statins can reduce CRP levels and independently decrease the risk of AF recurrence. Moreover, statins can control blood lipids and inflammatory factors such as IL-1, IL-6, and TNF-α [82, 83]. Studies have shown that infection with *H. pylori* may increase the risk of gastrointestinal bleeding and esophagitis in patients receiving anticoagulation therapy, underscoring the possible benefits of eradicating *H. pylori* in managing AF [84, 85]. Specifically, the simultaneous presence of *H. pylori* infection and the use of oral anticoagulants such as warfarin are associated with a heightened risk of developing peptic ulcers and subsequent gastrointestinal bleeding [86, 87, 88, 89]. Chronic *H. pylori*-associated gastritis is one of the main risk factors for gastrointestinal bleeding induced by oral anticoagulants; hence, eradicating *H. pylori* could significantly increase the safety of anticoagulant therapy in AF patients [90].

Nonetheless, these issues warrant additional investigation and research. The precise effects of *H. pylori* infection on the risk of gastrointestinal bleeding in patients receiving anticoagulation therapy remain unclear, necessitating further studies to fully comprehend the interactions between these two factors [91, 92].

Additionally, *H. pylori* infection and anticoagulant treatment may increase the incidence of iron deficiency anemia in female patients with AF, possibly because *H. pylori* affects iron absorption and anticoagulation therapy, leading to increased menstrual flow [93, 94, 95, 96]. Notably, the use of antibiotics such as clarithromycin could lead to digoxin toxicity, thereby affecting the AF treatment outcome; thus, medication interactions should be carefully managed to avoid exacerbating the condition [97, 98].

Despite studies suggesting that eradicating *H. pylori* may benefit the treatment of AF, given the potential drug interactions, treatment outcomes, and economic factors, there remains insufficient evidence to support the necessity of *H. pylori* eradication therapy in all AF patients. However, when AF patients infected with *H. pylori* experience high levels of inflammatory factors, abnormal blood lipids, and corresponding antibodies, it is beneficial to correct the possible sources of these risk factors and eradicate *H. pylori*. Therefore, extensive clinical research is required to explore this topic further.

## 6. Prognosis of Atrial Fibrillation in Relation to *H. pylori*

Chronic inflammation induced by *H. pylori* is closely linked to persistent and permanent AF onset and progression. Therefore, monitoring inflammation-related biomarkers, such as homocysteine levels, may be instrumental in predicting the severity and treatment outcomes of AF patients [58]. Some studies predict that adding the inflammatory factors IL-6 and CRP to the CHA2DS2-VASc risk score can improve its prognostic accuracy; however, excessive use of these inflammatory indicators in risk assessment can lead to incorrect anticoagulant use [39].

Additionally, using proton pump inhibitors may help reduce the recurrence of AF [37]. In contrast, low TGF-β1 levels may also be associated with AF recurrence, especially in patients infected with the Cag A strain. However, studies suggest that alterations in TGF-β1 may result from AF treatment rather than being a primary factor [15].

Moreover, *H. pylori* infection is considered a potential trigger for thrombogenesis, which could accelerate the formation of AF-related thrombi and even lead to myocardial infarction after catheter ablation. Among the antibodies produced against *H. pylori* by the human body, antibodies targeting *H. pylori* Vac A are recognized as the only causal factor linked to an increased risk of stroke, with CRP possibly facilitating this relationship. Therefore, targeting the CRP signaling pathway could lower the risk of stroke in patients infected with Vac A-positive strains of *H. pylori* [99, 100, 101]. Meanwhile, further research has indicated that eradicating *H. pylori* might decrease stroke risk in AF patients [99].

Additionally, the association of *H. pylori* with various cardiovascular diseases, such as coronary artery disease, heart failure, and other arrhythmias, could also influence the prognosis of AF patients, potentially leading to severe adverse outcomes [27, 102, 103, 104]. Therefore, the prognosis of AF patients is influenced not only by traditional cardiovascular risk factors but also by the potential effects of *H. pylori* infection. These findings suggest that considering the impact of *H. pylori* could be beneficial in managing AF patients, especially when assessing long-term prognosis and recurrence risk. However, controversy and limitations remain regarding the study of *H. pylori* in the prognosis of AF, meaning targeted research is needed to verify its true role.

## 7. Conclusion

Research has revealed a potential association between these two factors, suggesting that *H. pylori* may influence the onset and progression of AF through multiple mechanisms, including inflammation, metabolic dysregulation, immune responses, and changes in the gut microbiota. These mechanisms provide crucial insights into the treatment and prognosis of AF.

However, the causal relationship between *H. pylori* and AF remains unclear, and some studies have even failed to identify a definitive correlation. This inconsistency in research findings indicates that our current understanding remains incomplete and necessitates further clinical and experimental studies to explore the precise relationship between *H. pylori* and AF in depth. Hence, future research should focus on expanding sample sizes, increasing the number of randomized controlled trials, and conducting prospective studies to explore the interaction mechanism between *H. pylori* and AF. In addition, clinical studies can be performed to evaluate the potential benefits of *H. pylori* in improving the health outcomes of patients with AF.

In conclusion, while current evidence suggests a possible link between *H. pylori* and AF, research in this area is still in its infancy. Thus, comprehensive assessment and understanding of this complex relationship are crucial for developing effective treatment strategies and enhancing patient quality of life. Overall, exploring this topic promises to yield insights that could lead to significant advancements in managing both conditions.

## Abbreviations

AF, atrial fibrillation; C3, complement 3; C4, complement 4; CNS, central nervous system; DADs, delayed after-depolarizations; EADs, early after-depolarizations; ECGs, electrocardiograms; ENS, enteric nervous system; GBA, Gut-brain axis; *H. pylori*, *Helicobacter pylori*; HCY, homocysteine; HDL-C, high-density lipoprotein cholesterol; IL, interleukin; LDL, low density lipoprotein; MALT, mucosa-associated lymphoid tissue; NCX1, Na^+^/Ca^2+^ exchanger type-1; NCX, Na^+^/Ca^2+^ exchanger; SR, Sarcoplasmic reticulum; SCaEs, spontaneous SR Ca^2+^-release events; Th1, T helper 1 cell; Th2, T helper 2 cell; TCWs, Triggered Ca^2+^ Waves; TGF-β1, Transforming Growth Factor-beta1; Vac A, Vacuolating cytotoxin A.
